# Clinical Outcomes of Direct Oral Anticoagulants vs Warfarin for Extended Treatment of Venous Thromboembolism

**DOI:** 10.1001/jamanetworkopen.2023.28033

**Published:** 2023-08-15

**Authors:** Margaret C. Fang, Kristi Reynolds, Dongjie Fan, Priya A. Prasad, Sue Hee Sung, Cecilia Portugal, Elisha Garcia, Alan S. Go

**Affiliations:** 1 Division of Hospital Medicine, University of California, San Francisco; 2 Department of Research and Evaluation, Kaiser Permanente Southern California, Pasadena; 3 Department of Health System Science, Kaiser Permanente Bernard J. Tyson School of Medicine, Pasadena, California; 4 Division of Research, Kaiser Permanente Northern California, Oakland; 5 Department of Medicine, University of California, San Francisco; 6 Department of Epidemiology and Biostatistics, University of California, San Francisco; 7 Department of Medicine, Stanford University, Palo Alto, California

## Abstract

**Importance:**

Extending the duration of oral anticoagulation for venous thromboembolism (VTE) beyond the initial 3 to 6 months of treatment is often recommended, but it is not clear whether clinical outcomes differ when using direct oral anticoagulants (DOACs) or warfarin.

**Objective:**

To compare rates of recurrent VTE, hospitalizations for hemorrhage, and all-cause death among adults prescribed DOACs or warfarin whose anticoagulant treatment was extended beyond 6 months after acute VTE.

**Design, Setting, and Participants:**

This cohort study was conducted in 2 integrated health care delivery systems in California with adults aged 18 years or older who received a diagnosis of incident VTE between 2010 and 2018 and completed at least 6 months of oral anticoagulant treatment with DOACs or warfarin. Patients were followed from the end of the initial 6-month treatment period until discontinuation of anticoagulation, occurrence of an outcome event, health plan disenrollment, or end of the study follow-up period (December 31, 2019). Data were obtained from the Kaiser Permanente Virtual Data Warehouse and electronic health records. Data analysis was conducted from March 2022 to January 2023.

**Exposure:**

Dispensed prescriptions of DOACs or warfarin after a 6-month initial treatment for VTE.

**Main Outcomes and Measures:**

The primary outcomes were rates per 100 person-years of recurrent VTE, hospitalizations for hemorrhage, and all-cause death. Comparison of DOAC and warfarin outcomes were performed using multivariable Cox proportional hazards regression.

**Results:**

A total of 18 495 patients (5477 [29.6%] aged ≥75 years; 8973 women [48.5%]) with VTE who were treated with at least 6 months of anticoagulation were identified, of whom 2134 (11.5%) were receiving DOAC therapy and 16 361 (88.5%) were receiving warfarin therapy. Unadjusted event rates were lower for patients receiving DOAC therapy than warfarin therapy for recurrent VTE (event rate per 100 person-years, 2.92 [95% CI, 2.29-3.54] vs 4.14 [95% CI, 3.90-4.38]), hospitalizations for hemorrhage (event rate per 100 person-years, 1.02 [95% CI, 0.66-1.39] vs 1.81 [95% CI, 1.66-1.97]), and all-cause death (event rate per 100 person-years, 3.79 [95% CI, 3.09-4.49] vs 5.40 [95% CI, 5.13-5.66]). After multivariable adjustment, DOAC treatment was associated with a lower risk of recurrent VTE (adjusted hazard ratio [aHR], 0.66; 95% CI, 0.52-0.82). For patients prescribed DOAC treatment, the risks of hospitalization for hemorrhage (aHR, 0.79; 95% CI, 0.54-1.17) and all-cause death (aHR, 0.96; 95% CI, 0.78-1.19) were not significantly different than those for patients prescribed warfarin treatment.

**Conclusions and Relevance:**

In this cohort study of patients with VTE who continued warfarin or DOAC anticoagulation beyond 6 months, DOAC treatment was associated with a lower risk of recurrent VTE, supporting the use of DOACs for the extended treatment of VTE in terms of clinical outcomes.

## Key Points

**Question:**

Are there differences in clinical outcomes between treatment with direct oral anticoagulants (DOACs) and warfarin for people receiving extended oral anticoagulant treatment for venous thromboembolism (VTE)?

**Findings:**

In this cohort study of 18 495 adults with VTE who were prescribed anticoagulants for 6 or more months, DOAC treatment was associated with a lower risk of recurrent VTE. The risks for hospitalization for hemorrhage and all-cause death were not significantly different between patients taking DOACs vs warfarin.

**Meaning:**

These findings suggest that compared with warfarin, DOAC treatment is associated with a lower risk of recurrent VTE in patients taking extended anticoagulation treatment for VTE, supporting the use of DOACs for the extended treatment of VTE in terms of clinical outcomes.

## Teaser

This cohort study compares clinical outcomes for patients undergoing extended anticoagulation therapy with warfarin vs direct oral anticoagulants for treatment of venous thromboembolism (VTE).

## Introduction

Venous thromboembolism (VTE) is a common condition and a leading cause of sudden death.^1^ The primary treatment of VTE entails administering anticoagulation therapy for a period of 3 to 6 months during the acute active phase of thrombosis.^2^ Some patients may benefit from extending anticoagulation treatment duration beyond this initial treatment period, and current guidelines have recommended extended duration of anticoagulation treatment for selected patients, such as those with chronic persistent risk factors for VTE or if the VTE event occurred in the absence of a transient reversible risk factor (ie, unprovoked VTE).^2,3^

Options for extended treatment include vitamin K antagonists (predominantly warfarin) and the more recently developed direct oral anticoagulants (DOACs), which include the direct thrombin inhibitor dabigatran and factor Xa inhibitors. DOACs have been associated with lower bleeding risk than warfarin during the initial treatment phase of VTE.^4,5,6,7^ Less is known about the comparative outcomes of different anticoagulant options when used for extended VTE prevention, particularly beyond 12 months.^8,9,10^ Moreover, trials of DOACs for VTE excluded specific subgroups of patients, including those with chronic kidney disease or high bleeding risk. Because extended anticoagulation is associated with elevated bleeding risk, particularly in older patients, examining outcomes in these subgroups is important.^11,12^ The objective of this study was to compare the clinical outcomes of DOACs vs warfarin when used for the extended treatment of VTE, and assess whether there was heterogeneity of treatment outcomes by age, kidney function, and baseline bleeding risk.

## Methods

### Setting and Participants

This retrospective cohort study was approved by the institutional review boards of Kaiser Permanente Northern California (KPNC) and Kaiser Permanente Southern California (KPSC), and a waiver of informed consent was granted in accordance with 45 CFR § 46 because there was minimal risk for the study participants and because the research could not be practicably carried out without the waiver. We followed the Strengthening the Reporting of Observational Studies in Epidemiology (STROBE) reporting guideline for cohort studies. This study included adult members (aged ≥18 years) of KPNC and KPSC, 2 large, integrated health care delivery systems providing comprehensive care for more than 9 million health plan members across California. The source population is representative of the local and statewide population in California in terms of sociodemographics.^13,14^ Data for the study were obtained from the Kaiser Permanente Virtual Data Warehouse and included age, self-reported gender, self-reported race (including Asian or Pacific Islander, Black, White, other [Native American, Alaskan Native, and multiple races], and unknown) and Hispanic ethnicity, diagnoses codes, health care encounters, and pharmacy dispensing.^15^ Data on race and ethnicity were included to provide additional context about the population at risk.

Eligibility criteria were adults with a new diagnosis of acute VTE, defined as an incident clinical encounter with a primary or secondary diagnosis of VTE coded by the *International Classification of Diseases, Ninth Revision (ICD-9)* or *International Statistical Classification of Diseases and Related Health Problems, Tenth Revision (ICD-10)* during the sampling time period of January 1, 2010, to December 31, 2018, who were treated with an initial course of anticoagulants. Encounters could be from hospital, emergency department (ED), or outpatient settings. We then restricted the sample to individuals with complete information on age and gender who had at least 12 months of continuous enrollment and pharmacy benefits before the VTE index date. VTE type was categorized as pulmonary embolism with or without other thrombosis, lower extremity deep venous thrombosis, upper extremity deep venous thrombosis, and other VTEs of unusual sites (eg, splanchnic or cerebral sinus thrombosis). Superficial venous thrombosis and pregnancy-related VTE were not included.

### Anticoagulant Exposure

Medication information was obtained from health plan pharmacy dispensing records. We included only new users of oral anticoagulants, defined as people without a history of VTE and no prescription for warfarin or DOACs including dabigatran, rivaroxaban, apixaban, or edoxaban in the 12 months before the incident VTE diagnosis. For this study of extended anticoagulant therapy, the analysis focused on patients who had completed an initial course of treatment at least 6 months after the index VTE date (defined as 180 days of continuous anticoagulant exposure, with a grace period of ±10 days). A period of 6 months was chosen because 3 to 6 months is considered the standard duration of the primary treatment phase of initial VTE treatment,^2^ to match randomized trials of extended treatment, and because shorter durations could potentially include patients with transient risk factors for VTE, who generally have a lower baseline risk for VTE and do not benefit from extended anticoagulation.

We defined continuous anticoagulant therapy as the period covered by consecutive dispensed anticoagulant prescriptions, which was based on the number of days of medication supplied, prescription end date, and dispensed refill dates. We allowed for gaps of no more than 14 days between consecutive DOAC prescriptions and 30 days between warfarin prescriptions. Determination of continuous warfarin exposure also incorporated outpatient testing of international normalized ratios, as described in a previously validated algorithm.^16^ Permanent discontinuation of anticoagulant therapy was defined as occurring when there were 90 days or more without continuous anticoagulant exposure.

### Clinical Outcomes

The clinical outcomes for the study were rates per 100 person-years of recurrent VTE, hospitalization for hemorrhage, and all-cause death. We followed up patients for outcomes from the index VTE discharge date until discontinuation of anticoagulant therapy, disenrollment from the health plan, death, or the end of the study observation period (December 31, 2019). Recurrent VTE was defined as a new acute VTE encounter that occurred after the index VTE event. Potential events were identified from hospital, ED, and outpatient encounters with *ICD-9* and *ICD-10* diagnosis codes for VTE. For outpatient encounters, we also required the presence of a relevant radiology procedure (eg, chest computerized tomography angiogram or extremity ultrasonography) within 14 days. Because using diagnosis codes alone to identify VTE events results in many false-positive VTEs, we applied a rules-based algorithm based on diagnosis and procedure codes combined with natural language processing on unstructured electronic health records.^17,18^ This algorithm was internally developed and validated using a training set of 479 VTE events and had a positive predictive value of 95% and negative predictive value of 97% compared with physician review of medical records using standardized case definitions.

Hemorrhage events were identified using *ICD-9* and *ICD-10* discharge diagnosis codes. Intracranial hemorrhage was defined as a hospitalization with a primary or secondary discharge diagnosis of intracranial hemorrhage (intracerebral hemorrhage, subdural hematoma, and other intracranial hemorrhages such as subarachnoid or intraventricular hemorrhages). Hospitalizations for extracranial hemorrhages were based on primary discharge diagnosis codes. Deaths were identified using comprehensive data from health plan databases (including inpatient and ED deaths and proxy reports), state death certificate files, and the US Social Security Administration Death Master File.

### Clinical Variables

We specified covariates that could be confounders of anticoagulant choice and clinical outcomes. Categories of potential confounders were (1) factors associated with the risk of recurrent VTE (including history of cancer, hypercoagulable states, and other medical conditions associated with increased future risk of VTE), (2) factors associated with an increased risk of bleeding (eg, history of bleeding, kidney function, liver disease, and peptic ulcer disease), (3) comorbid conditions (including cardiovascular factors and overall comorbidity burden), (4) patient demographics (eg, age, and self-reported gender, race, and Hispanic ethnicity), and (5) medications (eg, long-term cardiovascular medications used as markers of underlying comorbidity and for risk adjustment purposes). Aspirin and nonsteroidal antiinflammatory drugs were not assessed due to their widespread availability without prescription. Cancer was determined from *ICD-9 and ICD-10* diagnosis codes, and diabetes was determined on the basis of diagnosis codes, laboratory data, and pharmacy data. Baseline serum, creatinine, and hemoglobin were defined as the most proximal outpatient laboratory value obtained in the past 3 years prior to the index VTE date.

*ICD-9* and *ICD-10* diagnosis codes were used to determine a baseline Charlson Comorbidity Index score^19^ for each patient. Baseline bleeding risk at the index VTE was calculated according to the RIETE (Registro Informatizado de Enfermedad Tromboembólica) bleeding risk estimation score^20^ and was categorized as low, intermediate, or high. Kidney function was classified according to the Chronic Kidney Disease Epidemiology Collaboration equation for estimated glomerular filtration rate (eGFR; defined as milliliters per minute per 1.73 m^2^)^21^ and using outpatient serum creatinine results from within 3 years and during the index VTE event. Long-term dialysis was identified by comprehensive health plan kidney failure treatment registries.

### Statistical Analysis

Our cohort comprised adults with acute VTE who took anticoagulants for at least 6 months and did not have a primary clinical outcome event during the initial treatment period. Anticoagulant type was based on initial prescription for DOACs or warfarin; the small number of patients (500 patients) who were dispensed both DOACs and warfarin during the initial treatment period were excluded from the analysis. We first described unadjusted rates of outcomes (recurrent VTE, hospitalizations for hemorrhage, and death). Next, we developed multivariable Cox regression models and adjusted for baseline characteristics of the cohort, which included sociodemographic variables, medical comorbidities, medications, index vital signs and selected laboratory values. All regression models also included a high-dimensional propensity score (HDPS) that represented the likelihood a patient was prescribed a DOAC or warfarin.^22^ The HDPS was developed using 5 dimensions (principal inpatient or ED diagnoses only, secondary inpatient or ED diagnoses, outpatient or ED diagnoses, procedures from any setting, and outpatient drug claims) and used a look-back period of up to 4 years. Within each dimension, the 300 most common codes were ranked by frequency. The final HDPS was then created by regressing the exposure variable (anticoagulation treatment type) on the covariates.

We then conducted 3 prespecified subgroup analyses to examine whether the association of anticoagulant type with outcomes varied by specific patient-level factors. Specifically, we focused on whether there was heterogeneity of treatment outcomes by patient age, kidney function, and baseline bleeding risk. For each subgroup, separate Cox regression models were developed, and heterogeneity of treatment outcomes was assessed using an interaction term between anticoagulant type and the subgroup of interest. All models were adjusted for baseline patient characteristics and an HDPS. When necessary, categories within individual subgroups were collapsed to allow for model convergence. Missing data were reported as a separate category for affected variables in the analysis. Significance levels were set at *P <* .05 with 2-sided, unpaired testing. Analyses were performed using SAS statistical software version 9.4 (SAS Institute) between March 2022 and January 2023.

## Results

We identified 18 495 patients (5477 [29.6%] aged ≥75 years; 8973 women [48.5%]) with VTE who were treated with at least 6 months of anticoagulation treatment (Figure). Most patients (16 361 patients [88.5%]) were prescribed warfarin and 2134 (11.5%) were prescribed a DOAC. Out of a total 3214.9 total person-years of DOAC exposure time, 2563.9 person-years (79.8%) were on dabigatran, 462.0 person-years (14.4%) were on rivaroxaban, and 188.9 person-years (5.9%) were on apixaban. No patients were prescribed edoxaban. DOACs were more commonly prescribed in later years (2016-2018) than earlier years (2010-2015), which is consistent with the expanding use of DOACs over time in the US (Table 1). By 2018, 58.5% of patients (1151 patients) with incident VTE were prescribed a DOAC instead of warfarin. On average, DOAC users continued to receive treatment longer than warfarin users (median [IQR] duration, 14.9 [8.7-22.2] months vs 10.8 [7.8-25.4] months) and were more likely to be younger, male, and non-Hispanic (Table 1). Patients prescribed DOACs were also more likely to have anticoagulants initiated in the ED and to have had a pulmonary embolism, but less likely to have prior cancer, kidney impairment, or high bleeding risk (Table 1). Missing data accounted for a small percentage overall. For example, the proportion of patients with missing data was 7.7% (1434 patients) for eGFR, 10.7% (1990 patients) for hemoglobin, 1.8% (339 patients) for body mass index (calculated as weight in kilograms divided by height in meters squared), and 1.2% (229 patients) for blood pressure.

**Figure.**
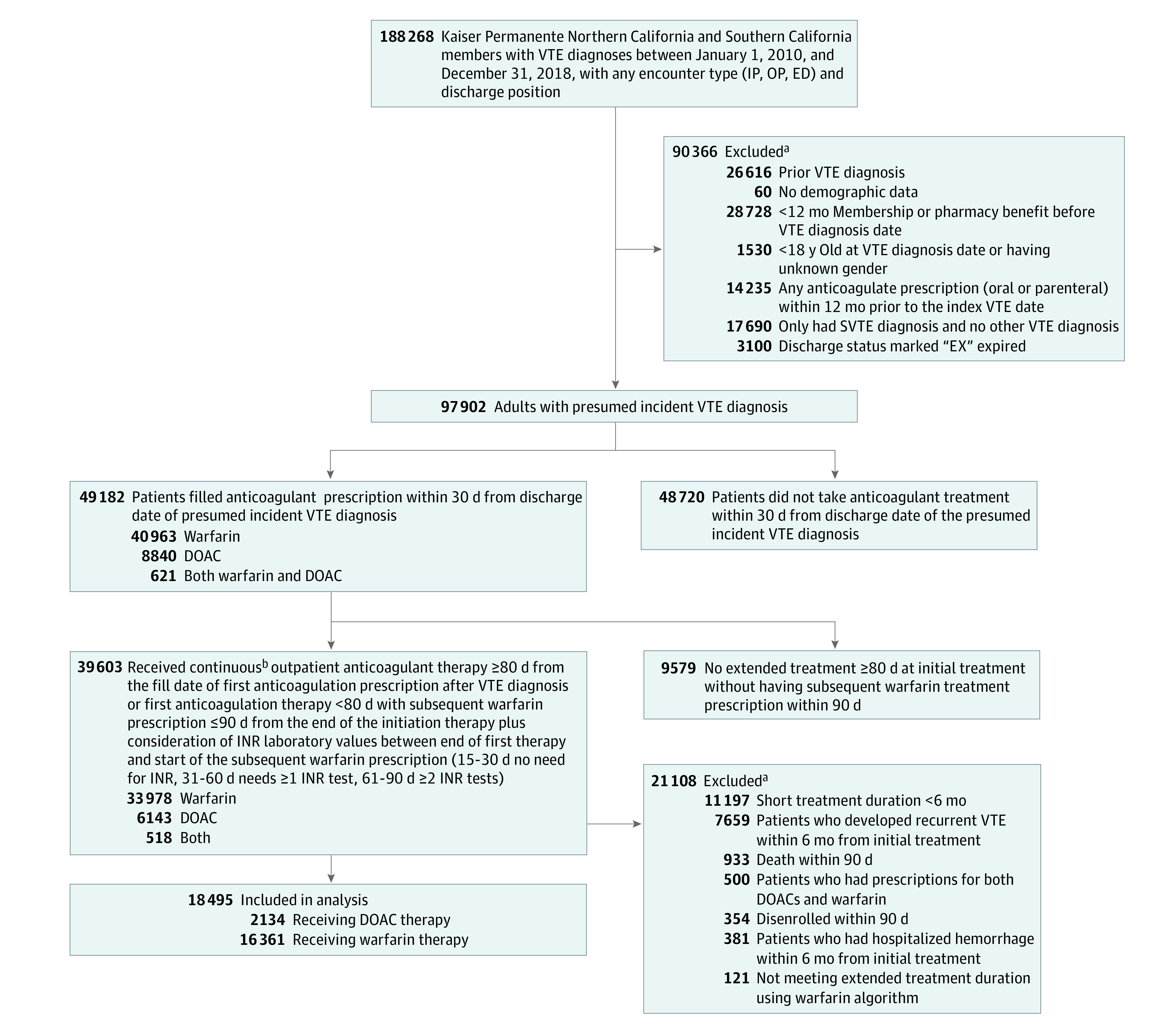
Cohort Assembly and Flowchart of 18 495 Patients With Venous Thromboembolism (VTE) Who Continued Anticoagulation Treatment for 6 Months or More DOAC indicates direct oral anticoagulant; ED, emergency department; INR, international normalized ratio; IP, inpatient; OP, outpatient; SVTE, superficial venous thromboembolism. ^a^Exclusion categories are not mutually exclusive (ie, some patients may have more than 1 exclusion). ^b^Continuous anticoagulant therapy is defined as 14 days or less of anticoagulation prescription gap for DOACs and 30 days or less of anticoagulation prescription gap for warfarin.

**Table 1.** Baseline Characteristics of Patients With VTE Who Received at Least 6 Months of Extended Anticoagulant Treatment With a DOAC or Warfarin

Characteristics	Patients, No. (%) (N = 18 495)		*P* value
	Extended treatment with DOAC (n = 2134)	Extended treatment with warfarin (n = 16 361)	
Anticoagulation treatment duration, median (IQR), mo	14.9 (8.7-22.2)	10.8 (7.8-25.4)	.001
DOAC^a^			
Dabigatran	1819 (85.2)	NA	NA
Rivaroxaban	410 (19.2)	NA	
Apixaban	158 (7.4)	NA	
Age group, y			
≤54	443 (20.8)	4085 (25.0)	<.001
55-64	431 (20.2)	3484 (21.3)	
65-74	619 (29.0)	3956 (24.2)	
75-84	459 (21.5)	3283 (20.1)	
≥85	182 (8.5)	1553 (9.5)	
Gender			
Women	982 (46.0)	7991 (48.8)	.01
Men	1152 (54.0)	8370 (51.2)	
Self-reported race			
Asian or Pacific Islander	95 (4.5)	766 (4.7)	.22
Black	309 (14.5)	2626 (16.1)	
White	1582 (74.1)	11 839 (72.4)	
Other^b^	17 (0.8)	180 (1.1)	
Unknown	131 (6.1)	950 (5.8)	
Self-reported Hispanic ethnicity	308 (14.4)	2747 (16.8)	.01
Setting of initial VTE diagnosis			
Outpatient	277 (13.0)	2595 (15.9)	<.001
Emergency department	1112 (52.1)	6568 (40.1)	
Hospital	745 (34.9)	7198 (44.0)	
VTE type			
Pulmonary embolism	1331 (62.4)	7748 (47.4)	<.001
Lower extremity deep vein thrombosis	665 (31.2)	6671 (40.8)	
Upper extremity deep vein thrombosis	66 (3.1)	993 (6.1)	
Other	30 (1.4)	433 (2.6)	
Not specified	42 (2.0)	516 (3.2)	
Baseline medical history			
Hypertension	1139 (53.4)	9542 (58.3)	<.001
Dyslipidemia	1373 (64.3)	10 295 (62.9)	.20
Former or current smoker	942 (44.1)	7228 (44.2)	.98
Diabetes	497 (23.3)	4088 (25.0)	.09
Chronic lung disease	560 (26.2)	4702 (28.7)	.02
Chronic kidney disease	406 (19.0)	3950 (24.1)	<.001
Diagnosed depression	446 (20.9)	3326 (20.3)	.54
Sepsis in hospital	209 (9.8)	1840 (11.2)	.04
Cancer	334 (15.7)	3014 (18.4)	.002
Coagulopathy	165 (7.7)	1213 (7.4)	.60
Diagnosed dementia	98 (4.6)	798 (4.9)	.56
Chronic liver disease	111 (5.2)	797 (4.9)	.51
Alcohol use disorder	110 (5.2)	432 (2.6)	<.001
Hypercoagulable hematologic conditions	53 (2.5)	468 (2.9)	.32
Inflammatory bowel disease	27 (1.3)	254 (1.6)	.31
Hospitalization for gastrointestinal hemorrhage	19 (0.9)	277 (1.7)	.01
Primary or secondary hypercoagulable state	43 (2.0)	257 (1.6)	.13
Hemiplegia or paraplegia	54 (2.5)	187 (1.1)	<.001
Hospitalization for nontraumatic intracranial hemorrhage	16 (0.7)	243 (1.5)	.01
Peptic ulcer disease	16 (0.7)	142 (0.9)	.58
Substance use disorder	38 (1.8)	88 (0.5)	<.001
Prior traumatic intracranial hemorrhage	9 (0.4)	119 (0.7)	.11
Hospitalization for other bleeding	7 (0.3)	76 (0.5)	.37
Mechanical fall	39 (1.8)	34 (0.2)	<.001
Hospitalization within 30 d	936 (43.9)	9367 (57.3)	<.001
Charlson Comorbidity Index score			
Low (<1)	1058 (49.6)	4820 (29.5)	
Mild (1-2)	688 (32.2)	5526 (33.8)	<.001
Moderate (3-4)	253 (11.9)	2911 (17.8)	
Severe (>4)	135 (6.3)	3104 (19)	
Length of hospital stay for index VTE, d			
Not hospitalized	1277 (59.8)	7728 (47.2)	<.001
<3	446 (20.9)	3012 (18.4)	
3-8	267 (12.5)	2938 (18)	
>8	144 (6.7)	2683 (16.4)	
Baseline estimated glomerular filtration rate, mL/min/1.73 m^2^			
≥90	457 (21.4)	3939 (24.1)	<.001
60-89	1095 (51.3)	6912 (42.2)	
45-59	289 (13.5)	2350 (14.4)	
30-44	100 (4.7)	1166 (7.1)	
15-29	17 (0.8)	348 (2.1)	
<15	0	86 (0.5)	
Dialysis or transplant	8 (0.4)	294 (1.8)	
Unknown	168 (7.9)	1266 (7.7)	
Body mass index^c^			
<18.5	27 (1.3)	231 (1.4)	.04
18.5-20.0	387 (18.1)	3144 (19.2)	
25.0-25.9	664 (31.1)	5117 (31.3)	
30.0-30.9	799 (37.4)	5721 (35.0)	
≥40	208 (9.7)	1858 (11.4)	
Unknown	49 (2.3)	290 (1.8)	
RIETE bleeding risk score at index VTE			
Low risk (0)	449 (21.0)	3968 (24.3)	<.001
Intermediate risk (1-4)	1658 (77.7)	12 055 (73.7)	
High risk (>4)	27 (1.3)	338 (2.1)	
Year of index VTE			
2010	0	1947 (11.9)	<.001
2011	0	2108 (12.9)	
2012	4 (0.2)	2132 (13.0)	
2013	1 (<0.0)	1938 (11.8)	
2014	5 (0.2)	2111 (12.9)	
2015	44 (2.1)	2174 (13.3)	
2016	244 (11.4)	1884 (11.5)	
2017	685 (32.1)	1252 (7.7)	
2018	1151 (53.9)	815 (5.0)	

Abbreviations: DOAC, direct oral anticoagulant; NA, not applicable; RIETE, Registro Informatizado de Enfermedad TromboEmbólica; VTE, venous thromboembolism.

^a^ Not mutually exclusive because patients could change from one DOAC to another during follow-up.

^b^ Other race included Native American, Alaskan Native, and multiple races.

^c^ Body mass index is calculated as weight in kilograms divided by height in meters squared.

During the follow-up period, we identified 1143 patients with recurrent VTEs, 530 patients with hospitalizations for hemorrhage, and 1601 deaths occurring among those taking extended warfarin treatment; and 84 patients with recurrent VTEs, 30 patients with hospitalizations for hemorrhage, and 112 deaths among those taking extended DOAC therapy. Compared with warfarin, DOAC treatment was associated with lower unadjusted rates of recurrent VTE (event rate per 100 person-years, 2.92 [95% CI, 2.29-3.54] vs 4.14 [95% CI, 3.90-4.38]), hospitalizations for hemorrhage (event rate per 100 person years, 1.02 [95% CI, 0.66-1.39] vs 1.81 [95% CI, 1.66-1.97]) and all-cause death (event rate per 100 person years, 3.79 [95% CI, 3.09-4.49] vs 5.40 [95% CI, 5.13-5.66]) (Table 2).

**Table 2.** Unadjusted Rates of Recurrent Venous Thromboembolism, Hospitalizations for Hemorrhage, and All-Cause Death Among Patients With Venous Thromboembolism While Receiving Extended Anticoagulant Treatment^a^

Event type and anticoagulant	Event rate/100 person-years (95% CI)
Recurrent venous thromboembolism	
Warfarin	4.14 (3.90-4.38)
Direct oral anticoagulant	
Any	2.92 (2.29-3.54)
Dabigatran	2.80 (2.12-3.49)
Rivaroxaban	3.18 (1.52-4.85)
Apixaban	3.88 (0.78-6.99)
Hospitalization for hemorrhage	
Warfarin	1.81 (1.66-1.97)
Direct oral anticoagulant	
Any	1.02 (0.66-1.39)
Dabigatran	1.12 (0.69-1.55)
Rivaroxaban	0.89 (0.02-1.77)
Apixaban	NA^b^
All cause-death	
Warfarin	5.40 (5.13-5.66)
Direct oral anticoagulant	
Any	3.79 (3.09-4.49)
Dabigatran	3.33 (2.59-4.07)
Rivaroxaban	6.02 (3.75-8.29)
Apixaban	4.30 (1.11-7.49)

Abbreviation: NA, not applicable.

^a^ Extended anticoagulant treatment is defined as 6 months or more of treatment.

^b^ There were no hospitalizations for hemorrhage among apixaban users; therefore, the rate was not calculated.

Multivariable models were all adjusted for baseline demographics, medical characteristics, laboratory tests, receipt of other cardiovascular medications, and an HDPS modeling the likelihood of DOAC prescription. After multivariable adjustment, DOACs continued to be associated with a significantly lower risk of recurrent VTE (adjusted hazard ratio [aHR] 0.66; 95% CI, 0.52-0.82). The associations of DOACs with risk of hospitalization for hemorrhage (aHR, 0.79; 95% CI, 0.54-1.17) and all-cause death (aHR, 0.96; 95% CI, 0.78-1.19) were not significantly different from those of warfarin (Table 3). We did not directly compare outcomes by individual DOACs due to the low numbers of patients prescribed rivaroxaban and apixaban in the cohort.

**Table 3.** Association of Anticoagulant Treatment With Clinical Outcomes in a Cohort of 18 495 Patients Receiving Extended Therapy for Venous Thromboembolism

Event type and treatment	aHR (95% CI)^a^
Recurrent venous thromboembolism	
DOAC	0.66 (0.52-0.82)
Warfarin	1 [Reference]
Hospitalization for hemorrhage	
DOAC	0.79 (0.54-1.17)
Warfarin	1 [Reference]
All-cause death	
DOAC	0.96 (0.78-1.19)
Warfarin	1 [Reference]

Abbreviations: aHR, adjusted hazard ratio; DOAC, direct oral anticoagulant.

^a^ Models were adjusted for baseline demographics, medical conditions, encounter type, medications, laboratory tests, and high-dimensional propensity score modeling likelihood of direct oral anticoagulant therapy.

Prespecified subgroup analyses were conducted to determine whether the association of anticoagulant type with outcomes differed by age group, kidney function, and baseline estimated bleeding risk (Table 4). In the analyses by age, the point estimates for recurrent VTE all favored DOAC treatment, although the comparison reached significance only in the subgroup of patients aged 75 years or older (HR, 0.49; 95% CI, 0.31-0.76). We did not find significant heterogeneity of treatment outcomes between age and anticoagulant type in any of the 3 clinical outcomes (Table 4). For the analysis of kidney function, the point estimates all favored DOAC therapy in risk of recurrent VTE, although the comparisons were not all statistically significant. The interaction term between treatment type and recurrent VTE was not significant (*P* for interaction = .37). There was significant heterogeneity of treatment outcomes in the test of treatment type and kidney function, where the HR of hospitalization for hemorrhage was 0.59 (95% CI, 0.35-1.00) in patients with eGFR greater than or equal to 60 mL/minute/1.73 m^2^ and 1.30 (95% CI, 0.71-2.39) in patients with eGFR less than 60 mL/minute/1.73 m^2^ (*P* for the interaction term between treatment type and kidney function = .03). This analysis did require collapsing the eGFR categories to achieve model convergence. In the subgroup analyses of bleeding risk, there were no significant differences in treatment effect size by baseline RIETE score (Table 4).

**Table 4.** Subgroup Analyses of Association of Anticoagulant Treatment Type with Clinical Outcomes

Event type and demographic	Direct oral anticoagulants vs warfarin, aHR (95% CI)^a^	*P* value for interaction
Recurrent venous thromboembolism		
Age, y		
≤54	0.54 (0.29-1.01)	.43
55-64	0.78 (0.47-1.27)	
65-74	0.80 (0.54-1.18)	
≥75^b^	0.49 (0.31-0.76)	
Kidney function (eGFR, mL/min/1.73 m^2^)		
≥90	0.67 (0.38-1.18)	.37
60-89	0.71 (0.52-0.97)	
45-59	0.65 (0.35-1.18)	
15-44 or kidney failure	0.29 (0.09-0.95)	
Unknown	0.76 (0.28-2.07)	
Bleeding risk (RIETE score)		
Low bleed risk (0)	0.66 (0.41-1.06)	.35
Intermediate to high bleed risk (≥1)^c^	0.65 (0.50-0.85)	
Hospitalization for hemorrhage		
Age, y		
≤54	0.95 (0.20-4.57)	.64
55-64	0.34 (0.10-1.19)	
65-74	0.75 (0.35-1.61)	
75-84	0.86 (0.42-1.74)	
≥85	1.16 (0.40-3.4)	
Kidney function (eGFR, mL/min/1.73 m^2^)		
≥60^d^	0.59 (0.35-1.00)	.03
<60 or kidney failure^d^	1.30 (0.71-2.39)	
Bleeding risk (RIETE score)		
Low bleed risk (0)	0.32 (0.04-2.73)	.27
Intermediate to high bleed risk (≥1)^c^	0.84 (0.56-1.26)	
All-cause death		
Age, y		
≤54	0.74 (0.14-3.76)	.16
55-64	1.39 (0.84-2.29)	
65-74	1.12 (0.76-1.65)	
75-84	0.81 (0.54-1.23)	
≥85	0.66 (0.41-1.06)	
Kidney function (eGFR, mL/min/1.73 m^2^)		
≥90	1.19 (0.67-2.11)	>.99
60-89	0.86 (0.64-1.17)	
45-59	0.92 (0.54-1.55)	
15-44 or kidney failure	1.10 (0.58-2.09)	
Unknown	1.12 (0.14-9.14)	
Bleeding risk (RIETE score)		
Low bleed risk (0)	1.62 (0.70-3.76)	.42
Intermediate bleed risk (1-4)	0.91 (0.73-1.14)	
High bleed risk (>4)	0.28 (0.01-7.59)	

Abbreviations: aHR, adjusted hazard ratio; eGFR, estimated glomerular filtration rate; RIETE, Registro informatizado de enfermedad tromboembólica.

^a^ Models for prespecified subgroup analyses were developed separately and included interaction terms between anticoagulant treatment type and subgroup of interest. All models adjusted for baseline demographics, medical conditions, encounter type, medications, laboratory tests, and high-dimensional propensity score modeling likelihood of direct oral anticoagulant therapy.

^b^ Age category collapsed to 75 years and older to allow for model convergence.

^c^ RIETE category collapsed to intermediate/high risk (≥1) to allow for model convergence.

^d^ eGFR categories collapsed to ≥60 and <60 mL/min/1.73 m^2^ to allow for model convergence.

## Discussion

In this cohort study of patients with VTE who continued warfarin or DOAC anticoagulation treatment beyond 6 months, DOAC treatment was associated with a lower risk of recurrent VTE. DOAC treatment was not associated with significantly different rates of hospitalizations for hemorrhage or death. In prespecified subgroup analyses, we did not find significant heterogeneity of treatment outcomes, except for a higher risk for hemorrhage associated with extended DOAC treatment among people with eGFR less than 60 mL/minute/1.73 m^2^.

Randomized trials^4,5,6,7^ comparing DOACs with warfarin for the initial treatment of VTE found DOACs to have generally better outcomes in terms of reducing recurrent VTE events. Trials^23,24,25^ of extended treatment with DOACs have largely compared DOACs with placebo. In 1 trial^25^ comparing dabigatran with warfarin, extended dabigatran therapy was noninferior to warfarin in preventing recurrent VTE, albeit with a numerically higher event rate. Our observational study of actual clinical care confirmed that DOACs (predominantly dabigatran) have favorable outcomes over warfarin beyond the initial treatment period. Our findings are consistent with an analysis^26^ of commercially insured patients that found apixaban, but not rivaroxaban, to be associated with a lower risk of hospitalization for recurrent VTE but without differences in hospitalization for bleeding or all-cause death. Our findings also support guidelines from the 2020 American Society of Hematology^2^ and the 2021 American College of Chest Physicians^3^ that suggest using DOACs over warfarin for extended VTE therapy.

More patients were prescribed warfarin than DOACs in our study, and the most frequently prescribed DOAC was dabigatran, reflecting the clinical practice in KPNC and KPSC during the study period. DOAC use did increase over time, most likely representing greater familiarity and availability of DOACs as first-line treatment for VTE. Our study could not comment on the relative safety or efficacy of individual DOACs. Factor Xa inhibitors, specifically apixaban, are increasingly prescribed in the US.^27,28^ Bleeding risk may also change over time with the recommendations for use of a reduced dose of apixaban and rivaroxaban for the secondary prevention of VTE after initial therapy.^2^ Further assessment of the relative safety and outcomes of extended reduced-dose factor Xa inhibitor therapy would be valuable, particularly in light of the concerning risks of bleeding while taking extended VTE therapy.^11^

We identified bleeding events in our study using an approach specific for major bleeding but less sensitive to minor bleeding,^29^ so the true rates of hemorrhage in our cohort may be higher than what we describe. However, the bleeding rates in our study (1.02 per 100 person-years with DOACs and 1.81 per 100 person-years with warfarin) were comparable with rates reported in a recent meta-analysis^11^ (1.12 per 100 person-years with DOACs and 1.74 per 100 person-years with warfarin) and in randomized trials of extended treatment with DOACs.^23,24,25^ A study strength was the inclusion of validated VTE events diagnosed and treated in outpatient settings, since VTE is commonly managed in an outpatient setting.^30^ We also used an approach to ascertain recurrent VTE that was more accurate than using diagnosis codes alone; diagnosis codes alone may falsely represent acute VTE as often as 60% of the time in outpatient encounters.^18^ We had access to comprehensive clinical and pharmacy dispensing data from integrated health care delivery systems in addition to natural language processing algorithms applied to electronic health record data, and could identify outcomes even if patients presented to facilities outside of KPNC or KPSC.

### Limitations

This study has limitations that should be acknowledged. Patients were not randomly assigned to warfarin or DOAC treatment, so unaccounted factors may have affected the choice of anticoagulant. For example, more people taking warfarin had severe chronic kidney disease and a higher Charlson Comorbidity Index score. We were also not able to ascertain whether there was differential use of nonprescription medications that may increase bleeding risk, such as aspirin or nonsteroidal antiinflammatory drugs. Although we applied HDPS approaches to control for baseline differences, it is possible that differential prescribing patterns contributed to observed differences in clinical outcomes. We did not know the planned duration of anticoagulation treatment for individual patients and were not able to determine the reasons for discontinuation of therapy. We chose to focus on patients who took anticoagulants for at least 6 months because patients at lower risk for VTE who have reversible or transient indications for anticoagulation are generally prescribed shorter durations of treatment. Discontinuation of anticoagulation treatment was inferred once there was a period of 90 consecutive days without evidence of anticoagulant exposure. Although this approach is likely to be specific to discontinuation of treatment, our analysis could have included the outcomes of patients who discontinued anticoagulation for shorter periods of time. In addition, although nearly one-half of patients in our study continued anticoagulants for more than 1 year, our study did not have many patients who continued anticoagulants for multiple years.

## Conclusions

In this cohort study of patients who completed at least 6 months of anticoagulation therapy for VTE, extended anticoagulation with DOACs, predominantly dabigatran, was associated with a lower risk for recurrent VTE compared with extended warfarin treatment. Our study contributes to the growing evidence supporting the use of DOACs for both initial and extended treatment of VTE in terms of clinical outcomes as well as treatment satisfaction.^31^ Future investigations should address the comparative outcomes of a reduced dose of DOACs for VTE as well as the optimal duration of treatment for individual patients.

## Notes

Supplement.
Data Sharing Statement
