# Supplementary material for: Clinical Outcomes of Direct Oral Anticoagulants vs Warfarin for Extended Treatment of Venous Thromboembolism
Source: JAMA Netw Open. 2023 Aug 15;6(8):e2328033. doi: 10.1001/jamanetworkopen.2023.28033 (PMC10427945; doi:10.1001/jamanetworkopen.2023.28033)
Supplement: Supplement. — Data Sharing Statement [file jamanetwopen-e2328033-s001.pdf]

Data Sharing Statement

Fang. Clinical Outcomes of Direct Oral Anticoagulants vs Warfarin for Extended Treatment of Venous Thromboembolism. *JAMA Netw Open*. Published August 15, 2023. doi:10.1001/jamanetworkopen.2023.28033

Data
Data available: No
